# Anatomic prognostic factors and their potential roles in refining M1 classification for de novo metastatic nasopharyngeal carcinoma

**DOI:** 10.1002/cam4.6816

**Published:** 2023-12-11

**Authors:** Tian‐Zhu Lu, Fu‐juan Zeng, Yu‐Jun Hu, Min Fang, Fang‐yan Zhong, Bi‐juan Chen, Hao Zhang, Qiao‐juan Guo, Jian‐ji Pan, Xiao‐chang Gong, Shao Hui Huang, Zhao‐hui Liao, Yunfei Xia, Jin‐gao Li

**Affiliations:** ^1^ NHC Key Laboratory of Personalized Diagnosis and Treatment of Nasopharyngeal Carcinoma Jiangxi Clinical Research Center for Cancer, Jiangxi Cancer Hospital, The Second Affiliated Hospital of Nanchang Medical College Nanchang Jiangxi China; ^2^ Department of Radiation Oncology Jiangxi Clinical Research Center for Cancer, Jiangxi Cancer Hospital, The Second Affiliated Hospital of Nanchang Medical College Nanchang Jiangxi China; ^3^ State Key Laboratory of Oncology in South China, Collaborative Innovation Center for Cancer Medicine Sun Yat‐sen University Cancer Center Guangzhou China; ^4^ Department of Radiation Oncology Sun Yat‐sen University Cancer Center Guangzhou China; ^5^ Department of Radiation Oncology Fujian Medical University Cancer Hospital & Fujian Cancer Hospital Fuzhou China; ^6^ Department of Radiation Oncology, Hubei Cancer Hospital, Tongji Medical College Huazhong University of Science and Technology Wuhan China; ^7^ Department of Radiation Oncology, Princess Margaret Cancer Centre University of Toronto Toronto Ontario Canada; ^8^ Nursing Education Training Center Jiangxi Clinical Research Center for Cancer, Jiangxi Cancer Hospital, The Second Affiliated Hospital of Nanchang Medical College Nanchang Jiangxi China

**Keywords:** immunotherapy, M1 categories, nasopharyngeal carcinoma, oligometastatic disease, radiotherapy

## Abstract

**Background and Purpose:**

To identify anatomic prognostic factors and their potential roles in refining M1 classification for de novo metastatic nasopharyngeal carcinoma (M1‐NPC).

**Materials and Methods:**

All M1‐NPC treated with chemotherapy and/or radiotherapy between 2010 and 2019 from two centers (training and validation cohort) were included. The prognostic value of metastatic disease extent and involved organs for overall survival (OS) were assessed by several multivariable analyses (MVA) models. A new M1 classification was proposed and validated in a separate cohort who received immuno‐chemotherapy.

**Results:**

A total of 197 M1‐NPC in the training and 307 in the validation cohorts were included for M1 subdivision study with median follow‐up of 46 and 57 months. MVA model with “≤2 organs/≤5 lesions” as the definition of oligometastasis had the highest C‐index (0.623) versus others (0.606–0.621). Patients with oligometastasis had better OS versus polymetastasis (hazard ratio [HR] 0.47/0.63) while liver metastases carried worse OS (HR 1.57/1.45) in MVA in the training/validation cohorts, respectively. We proposed to divide M1‐NPC into M1a (oligometastasis without liver metastases) and M1b (liver metastases or polymetastasis) with 3‐year OS of 66.5%/31.7% and 64.9%/35.0% in the training/validation cohorts, respectively. M1a subset had a better median progress‐free survival (not reach vs. 17 months, p < 0.001) in the immuno‐chemotherapy cohort (*n* = 163).

**Conclusion:**

Oligometastasis (≤2 organs/≤5 lesions) and liver metastasis are prognostic for M1‐NPC. Subdivision of M1‐NPC into M1a (oligometastasis without liver metastasis) and M1b (liver metastasis or polymetastasis) depicts the prognosis well in M1‐NPC patients who received immuno‐chemotherapy.

## 1 INTRODUCTION

De novo metastatic nasopharyngeal carcinoma (M1‐NPC) accounts for 5%–10% of newly diagnosed NPC cases.^1^ M1‐NPC generally has a poor long‐term survival, with 3‐year overall survival (OS) of 20%–30%.^2^, ^3^, ^4^ However, heterogeneity exist. With contemporary treatment, a subset of M1‐NPC patients is able to enjoy long‐term survival or even cure.^5^, ^6^, ^7^, ^8^

Current M classification of the eighth edition TNM (TNM‐8) has classified all M1 NPC as one stage group without further sub‐categorization. Emerging data showed that M1‐NPC patients with different metastatic extent or site of metastasis may have different outcomes and may require different treatment approaches.^8^, ^9^, ^10^, ^11^, ^12^ OMD is reported as an important prognostic indicator for M1‐NPC.^4^, ^6^, ^7^, ^13^ However, the definition of OMD remains unsettled. Some studies divide OMD and polymetastatic disease (PMD) according to number of metastatic lesions,^7^, ^14^, ^15^ while others also included number of metastatic organs.^16^, ^17^ In addition, various metastatic organs appear to have differential prognostic importance. It has been reported that liver metastases carried worse OS versus other metastases, while NPC patients with lung metastases have a better prognosis.^8^, ^12^ These data suggest that OMD/PMD and the presence/absence of liver metastasis have the potential to refine current M1 classification, and to further guide treatment in M1‐NPC.

Retrospective and prospective clinical studies have confirmed that locoregional radiotherapy (LR‐RT), that is, RT to primary tumor and cervical nodal areas, in M1‐NPC can significantly improve progression‐free survival (PFS) and OS.^18^, ^19^ However, previous retrospective studies have confirmed that not all patients with M1‐NPC benefit from LR‐RT and that OMD status may be an excellent way to differentiate the benefiting population.^4^, ^13^, ^20^ In addition, systemic treatment of M1‐NPC has entered a new era of immunotherapy. Anti‐PD‐1 monoclonal antibody (mAb) combined with chemotherapy is now considered the first‐line standard‐of‐care.^21^, ^22^, ^23^ Therefore, deriving M1 subcategories that can effectively depict prognosis following immuno‐chemotherapy is worthy of exploration.

Here, we used data from two academic centers in NPC‐endemic areas (one served as the training and the other as the validation cohort) to assess the prognostic value of metastatic disease extent (OMD vs. PMD) and involved organ(s) (liver vs. lung vs. bone vs. other) in M1‐NPC. We hope to derive a prognostically most appropriate OMD definition to identify a subset who may benefit from LR‐RT, and to propose a refined M classification that could depict prognosis well in M1‐NPC following contemporary immuno‐chemotherapy.

## 2 MATERIALS AND METHODS

### 2.1 Patient cohorts

Patients with M1‐NPC diagnosed and treated at Jiangxi Cancer Hospital from January 2010 to December 2019 were used as the training cohort while validation cohort was derived by randomly selection of 25% of M1‐NPC patients (*n* = 1400) treated at the Sun Yat‐sen University Cancer Center from January 2010 to December 2019. The inclusion criteria of the two cohorts were as follows: (i) pathologically‐proven NPC, (ii) pathological or imaging diagnosis of de novo distant metastatic disease at diagnosis, and (iii) received at least two cycles of first‐line chemotherapy. The exclusion criteria were as follows: (i) prior radiotherapy in the head and neck region, and (ii) history of other malignancies in the past 5 years.

In addition, an immuno‐chemotherapy cohort was assembled which included M1‐NPC patients treated with anti‐PD‐1 mAb combined with chemotherapy as the first‐line treatment between January 2018 and July 2021 at four cancer centers (Sun Yat‐Sen University Cancer Center, Jiangxi Cancer Hospital, Fujian Cancer Hospital, and Hubei Cancer Hospital). The inclusion criteria were as follows: (i) pathologically proven NPC, (ii) pathological or imaging diagnosis of distant metastasis, and (iii) received at least two cycles of chemotherapy plus anti‐PD‐1 mAb. The exclusion criteria were: history of other malignant tumors in the past 5 years.

To complete the staging diagnosis, flexible fiberoptic endoscopy, MRI of the head and neck, basic serum chemistry, EBV‐DNA, chest CT, bone scan and ultrasound/CT of liver and abdomen, or positron emission tomography/computed tomography (PET/CT) were included. If liver metastasis was found by B‐ultrasound, supplementary examinations with enhanced CT or enhanced MRI was conducted. If bone metastases are possible on ECT, enhanced MRI was generally completed. Each institution's ethics review board approved the study, and exempted informed consent for the collection of clinical data from patients.

### 2.2 Treatment

Patients received at least two cycles of platinum‐based chemotherapy (once in every 3 weeks). After chemotherapy, LR‐RT was considered according to the treatment plan. Intensity‐modulated radiation therapy (IMRT) was used to treat nasopharyngeal lesions and regional metastatic lymph nodes using a cumulative dose of 66–70 Gy/30–35 fractions. Patients received anti‐PD‐1 monoclonal antibodies, including camrelizumab, toripalimab, tislelizumab, pembrolizumab, penpulimab, sintilimab, and nivolumab (all 200 mg intravenously on Day 1, except toripalimab, which was 240 mg intravenously on Day 1). All anti‐PD‐1 monoclonal antibodies were administered every 3‐week cycle for at least two cycles until tumor progression, intolerable side effects, or the doctor's decision. Detailed information and the dose of chemotherapy regimens for patients are provided in the Supplementary Methods.

### 2.3 Recording metastatic organs and metastatic lesions

Liver, lung, bone, distant lymph nodes (excluding cervical lymph node metastases), spleen, adrenal glands, and meningeal metastases were counted as one metastatic organ each. Number of lesions was counted based on imaging findings while distant metastatic lymph nodes were counted as one lesion per each individual lymphatic drainage area (axillary, mediastinum, retroperitoneal, and inguinal).

### 2.4 Statistical analyses

The study comprised 6 steps:

1. Step 1. Identifying potential anatomic prognostic factors in univariable analysis (UVA)
2. Step 2. Deriving an appropriate definition of OMD versus PMD based on highest C‐index among various multivariable analysis (MVA) models
3. Step 3. Assessing the prognostic importance of liver versus other organ metastases
4. Step 4. Proposing a refined M classification that further subcategorized M1‐NPC into M1a and M1b
5. Step 5. Confirming the value of M1a versus M1b subdivision in predicting who might benefit from LR‐RT
6. Step 6. Validating the ability of M1a and M1b subdivision in depicting outcomes in a separate multi‐center cohort of M1‐NPC treated with immuno‐chemotherapy

Chi‐squared or Fisher's exact tests were used to compare categorical variables, and the Mann–Whitney *U*‐test was used to compare continuous variables. Actuarial rates of OS (any cause of death as an event) and PFS (disease progression or death as an event) were calculated using the Kaplan–Meier method with log‐rank test for comparison of different groups. All time‐to events were calculated from the first diagnosis of M1‐NPC. UVA and MVA were performed using the Cox proportional hazard model to assess the prognostic value of metastatic organs (bone, liver, lung, or other metastases), number of metastatic organs, and number of the metastatic lesions, together with gender, age, ECOG performance status, T categories, N categories, LR‐RT, local treatment for metastatic lesions, Chemotherapy, Epstein–Barr virus (EBV) DNA (Low vs. High), and lactate dehydrogenase (LDH) (Normal vs. Abnormal). Harrell's C‐index (C‐index) was used to compare the predictability of OS for MVA models included various definition of OMD. All tests were two‐sided with a *p* < 0.05 as statistically significance.

## 3 RESULTS

### 3.1 Baseline characteristics

Of 340 M1‐NPC patients treated in Jiangxi Cancer Hospital during the study period, 79 did not complete two cycles of palliative chemotherapy, 24 lacked complete radiological data, 37 received 2D routine radiotherapy, and 3 had other primary malignancies. The remaining 197 patients were included as the training cohort while 307 cases of M1‐NPC from the Sun Yat‐sen University Cancer Center were included as the validation cohort (Figure S1).

Bone, liver, lung, and distant lymph nodes or metastases at other sites were identified in 64.5%, 36.0%, 24.4%, and 15.7% of patients in the training cohort, and 76.9%, 30.6%, 23.8%, and 21.8% of patients in the validation cohort, respectively. Moreover, 64.0% of patients in the training cohort received LR‐RT, and 155 (50.5%) in the validation cohort received LR‐RT. Detailed information is shown in Table 1.

**TABLE 1:** Clinical characteristics of patients in training and validation cohorts.

Variables	Training cohort, *n* (%)	Validation cohort, *n* (%)	*p*
Case Number	197	307	
Gender			
Male	164 (83.2)	258 (84.0)	0.815
Female	33 (16.8)	49 (16.0)	
Age, median (IQR; years)	52 (43–59)	46 (39–54)	<0.001
ECOG			
0–1	189 (95.9)	298 (97.1)	0.493
2	8 (4.1)	9 (2.9)	
T categories^a^			
T1‐3	123 (62.4)	191 (62.2)	0.960
T4	74 (37.6)	116 (37.8)	
N categories^a^			
N0‐1	36 (18.3)	67 (21.8)	0.281
N2‐3	161 (81.7)	240 (78.2)	
Bone metastases			
None	70 (35.5)	84 (27.4)	0.052
Yes	127 (64.5)	223 (72.6)	
Liver metastases			
None	122 (64.0)	213 (69.4)	0.084
Yes	75 (36.0)	94 (30.6)	
Lung metastases			
None	149 (75.6)	234 (76.2)	0.880
Yes	48 (24.4)	73 (23.8)	
Other metastases			
None	166 (84.3)	240 (78.2)	0.920
Yes	31 (15.7)	67 (21.8)	
LR‐RT			
None	71 (36.0)	152 (49.5)	0.003
Yes	126(64.0)	155 (50.5)	
Local treatment for metastatic lesions			
None	158 (80.2)	257 (83.7)	0.313
Yes	39 (19.8)	50 (16.3)	
Local treatment method for metastatic lesions			
None	158 (80.2)	257 (83.7)	0.148
Radiotherapy for metastatic	36 (18.3)	43 (14.0)	
Radiofrequency ablation for liver metastases	3 (1.5)	7 (2.3)	
Chemotherapy			
<6 cycles	124 (62.9)	107 (34.9)	<0.001
≥6 cycles	73 (37.1)	200 (65.1)	
EBV DNA			
Undetectable	44 (22.3)	37 (12.1)	0.006
Detectable	143 (72.6)	258 (84.0)	
NA	10 (5.1)	12 (3.9)	
EBV‐DNA(Copies/mL)^c^			
Best cut‐off	4700	11,800	0.001
Low	90 (48.1)	98 (33.2)	
High	97 (51.9)	197 (66.8)	
LDH(IU/L)			
Normal	131 (66.5)	187 (60.9)	0.205
Abnormal^b^	66 (33.5)	120 (39.1)	
No. of metastatic organ			
1	135 (68.5)	204 (66.4)	0.299
2	41 (20.8)	56 (18.2)	
≥3	21 (10.7)	47 (15.3)	
No. of metastatic lesion			
1	48 (24.4)	83 (27.0)	0.228
2	20 (10.1)	37 (12.1)	
3	17 (8.6)	32 (10.4)	
4	23 (11.7)	17 (5.5)	
5	11 (5.6)	19 (6.2)	
>5	78 (39.6)	119 (38.8)	

^a^ According to the 8th edition of the American Joint Committee on Cancer/ Union for International Cancer Control cancer staging manual.

^b^ Abnormal threshold, >250 U/L.

^c^ Detectable thresholds: 0 copy/mL.

Abbreviations: EBV DNA, Epstein–Barr virus deoxyribonucleic acid; IQR, interquartile range; LDH, Lactate dehydrogenase; LR‐RT, locoregional radiotherapy.

### 3.2 Potential anatomic prognostic factors in UVA

**FIGURE 1:**
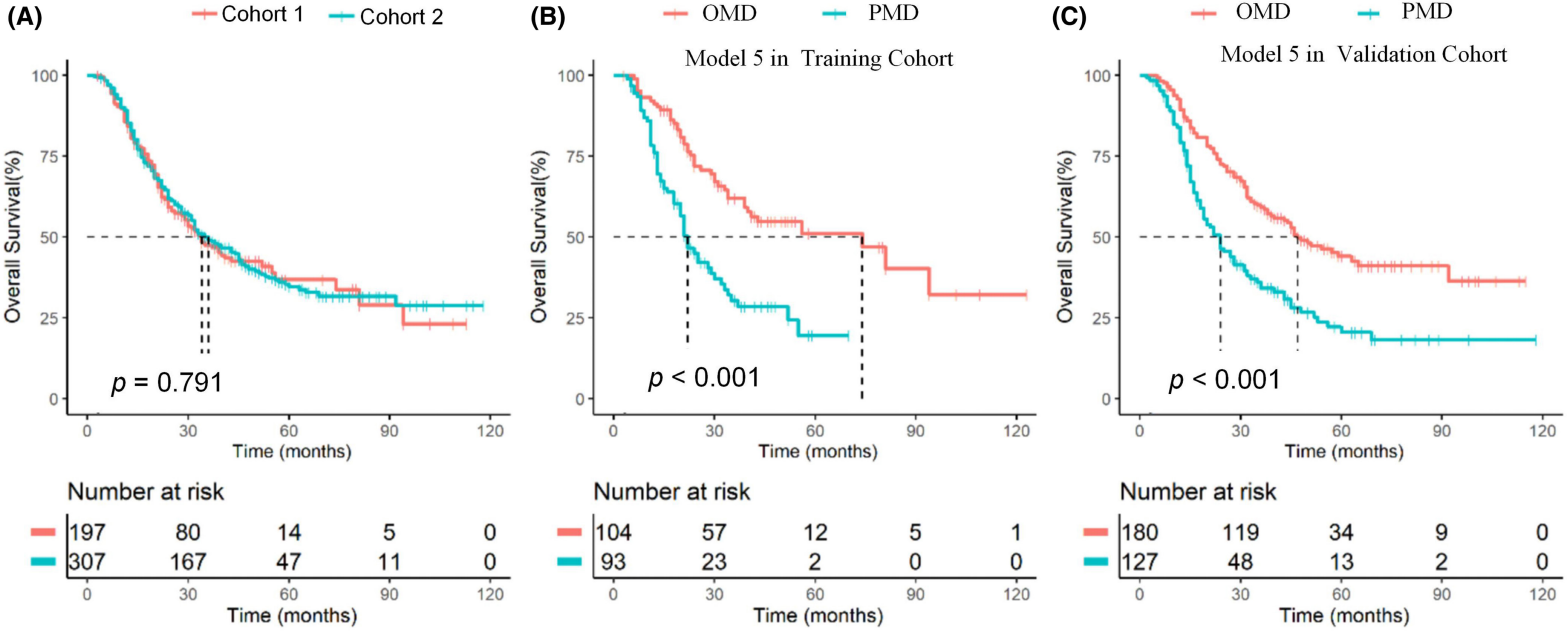
OS in the training versus validation cohorts (A), and stratified by oligometastasis versus polymetastasis (Model 5) in the training (B) and validation cohorts (C). OS, overall survival; OMD, oligometastatic disease; PMD, polymetastatic disease.

The median follow‐up of the training and validation cohort was 45 and 57 months, and the 3‐years OS of the training and validation cohort was 47.1% and 49.0%, respectively (*p* = 0.791, Figure 1A). UVA showed that liver metastases [training: Hazard ratio (HR) 1.83, (95% confidence interval: 1.24–2.72), *p* = 0.003; validation: HR 1.75 (1.29–2.36), *p* < 0.001], number of metastatic organs [training: HR 1.97 (1.31–2.95), *p* = 0.001; validation: HR 1.53 (1.13–2.06), *p* = 0.006], number of metastatic lesions [training: HR 2.56 (1.72–3.81), *p* < 0.001; validation: HR 2.02 (1.51–2.71), *p* < 0.001] were potential anatomic prognostic factors, together with EBV DNA [training: HR 1.76 (1.07–2.88), *p* = 0.025; validation HR 1.69 (1.21–2.36, *p* = 0.002], LDH [training HR 2.11 (1.41–3.14), *p* < 0.001; validation: HR 1.39 (1.03–1.87), *p* = 0.029], and LR‐RT [training: HR 0.44 (0.30–0.66, *p* < 0.001; validation: HR 0.54 (0.35–0.83), *p* < 0.001] (Table S1).

### 3.3 Derivation of a most appropriate definition of oligometastasis with MVA

The following six MVA models, based on different combination of the number of metastatic organs and lesions commonly used in literature for OMD definition, were constructed to derive a prognostically most appropriate differentiation of OMD versus PMD: Model 1 (one metastatic lesion), Model 2 (one metastatic organ and ≤3 metastatic lesions), Model 3 (one metastatic organ and ≤5 metastatic lesions), Model 4 (≤2 metastatic organs and ≤3 metastatic lesions), Model 5 (≤2 metastatic organs and ≤5 metastatic lesions), and Model 6 (≤3 metastatic organs and ≤5 metastatic lesions). OMD had higher OS versus PMD in all six models (all *p* < 0.001; Figure 1B,C and Figures S2 and S3). The C‐indices of the six models were 0.613/0.606/0.618/0.617/0.623/0.621 and 0.563/0.585/0.592/0.575/0.593/0.591 in the training and validation cohorts, respectively (Table 2). Model 5 (≤2 metastatic organs and ≤5 metastatic lesions) had the highest performance in OS prediction, and we consider it as the most appropriate OMD definition for M1‐NPC. The median OS of patients with OMD was significantly better than that of patients with PMD in both the training and validation cohorts (both *p* < 0.001; Figure 1B,C and Figure S4).

**TABLE 2:** The C‐index and AIC of various MVA models to define OMD.

OMD Models	Training cohort	Validation cohort
Model 1 (one metastatic lesion)	0.613	0.563
Model 2 (one metastatic organ and ≤3 metastatic lesions)	0.606	0.585
Model 3 (one metastatic organ and ≤5 metastatic lesions)	0.618	0.592
Model 4 (≤ 2 metastatic organs and ≤3 metastatic lesions)	0.617	0.575
Model 5 (≤ 2 metastatic organs and ≤5 metastatic lesions)	0.623	0.593
Model 6 (≤ 3 metastatic organs and ≤5 metastatic lesions)	0.621	0.591

Abbreviations: C‐index, concordance index; OMD, oligometastatic disease.

After adjusting for sex, age, ECOG status, T category, N category, number of chemotherapy cycles, LR‐RT, local treatment for metastatic lesions, EBV DNA, and LDH, MVA showed that OMD was an independent prognostic factor in the training cohorts (HR 2.11 [1.37–3.25], *p* = 0.001), which was further verified in the validation cohort (HR 1.60 [1.17–2.18], *p* = 0.003) (Table 3). Note that liver metastasis status and OMD were not simultaneously included in the MVA model, given their certain collinearity.

**TABLE 3:** Multivariable analysis of OS in M1‐NPC patients.

Variables	Training cohort		Validation cohort	
	HR (95%CI)	*p*	HR (95%CI)	*p*
MAV analyses include OMD				
OMD	0.47 (0.31–0.73)	0.001	0.63 (0.46–0.85)	0.003
EBV DNA	1.68 (1.08–2.61)	0.021	1.44 (1.02–2.03)	0.037
LR‐RT	0.69 (0.44–1.08)	0.106	0.57 (0.42–0.77)	<0.001
MAV analyses included metastatic organ				
Liver metastases	1.57 (1.04–2.38)	0.032	1.45 (1.05–2.01)	0.023
EBV DNA	1.66 (1.07–2.58)	0.024	1.51 (1.07–2.12)	0.018
LR‐RT	0.60 (0.39–0.94)	0.025	0.57 (0.42–0.78)	<0.001

Abbreviations: M1‐NPC, de novo metastatic nasopharyngeal carcinoma; OMD, oligometastatic disease; LR‐RT, locoregional radiotherapy; EBV DNA, Epstein–Barr virus deoxyribonucleic acid.

### 3.4 Confirmation of the prognostic value of liver metastasis in MVA

Compared to other organ metastasis, liver metastases had a significantly worse 3‐year OS (35.2% vs. 55.0%, *p* < 0.001 and 35.8% vs. 54.6%, *p* < 0.001, Figure S3A,B). MVA showed that among the metastatic organs, only liver metastasis was an independent prognostic factor for M1‐NPC in the training (HR 1.57 [1.04–2.38], *p* = 0.032) and validation cohorts (HR 1.45 [1.05–2.01], *p* = 0.023) (Table 3).

### 3.5 Proposal of subdivision of M1 in to M1a and M1b subcategories

**FIGURE 2:**
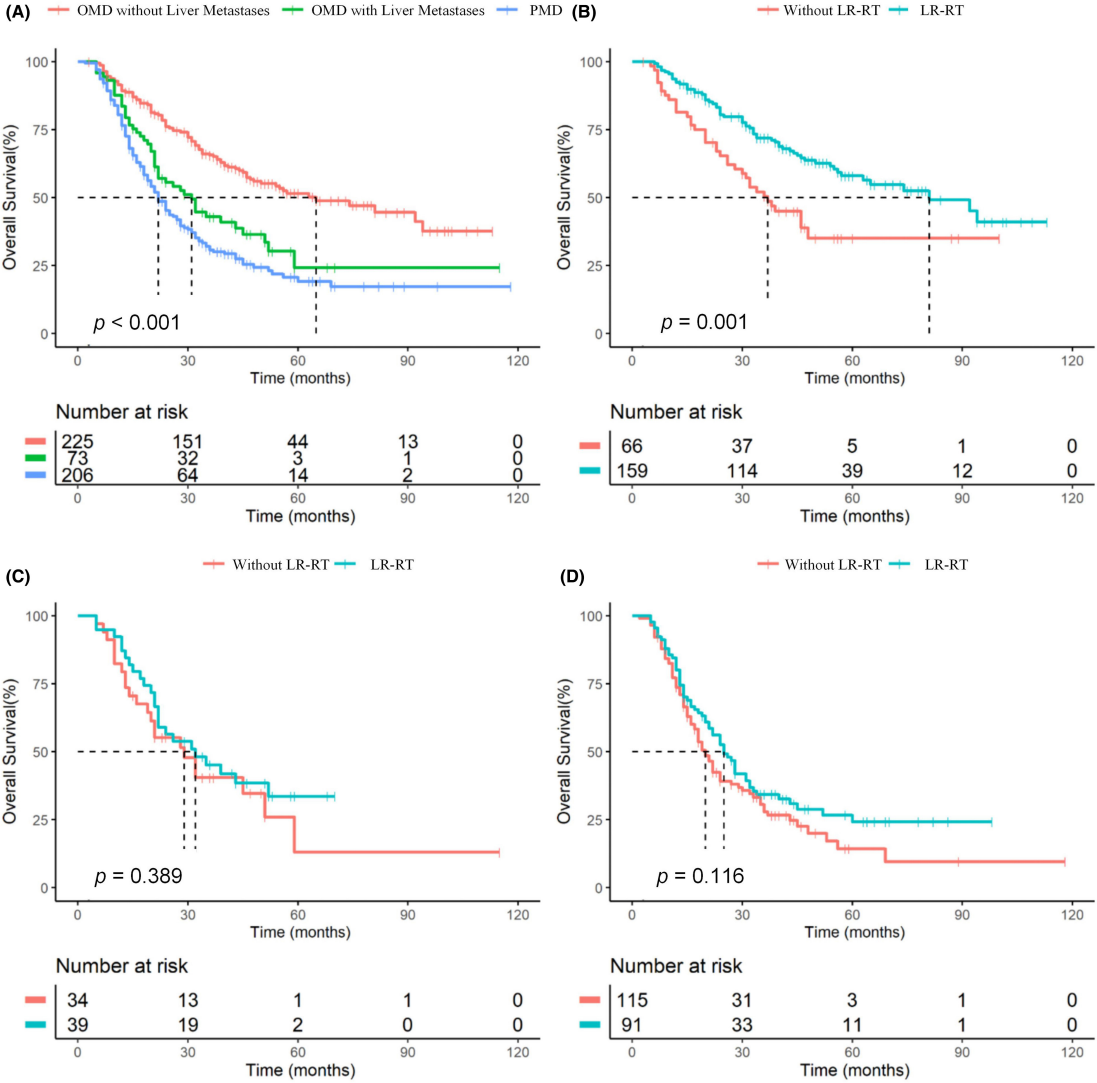
OS stratified by OMD with versus OMD without liver metastases versus PMD Groups (A); OS stratified by with versus without LR‐RT in OMD without liver metastasis (B), OMD with liver metastases (C), and PMD (D) subgroups. OS, overall survival; OMD, oligometastatic disease; PMD, polymetastatic disease; LR‐RT: locoregional radiotherapy.

Considering that OMD status and liver metastases were both important prognostic factors for M1‐NPC, we combined data from both cohorts to refine the M1 subgroup. We constructed a subcategory model to classify the patients into three groups: A (OMD without liver metastases), B (OMD with liver metastases), and C (PMD). Kaplan–Meier survival analysis showed that the 3‐year OS of A, B, and C in the training cohort were 65.6%, 42.2%, and 33.1%, respectively (*p* < 0.001, Figure 2A). In group A, 3‐year OS was significantly better in patients who received LR‐RT than in those without LR‐RT (71.4% vs. 50.5%, *p* = 0.001, Figure 2B), while in groups B and C, there was no significant difference in the 3‐year OS between those who received LR‐RT and those without LR‐RT (44.2% vs. 38.6%, *p* = 0.389, Figure 2C; 33.8% vs. 27.9%, *p* = 0.116, Figure 2D).

**FIGURE 3:**
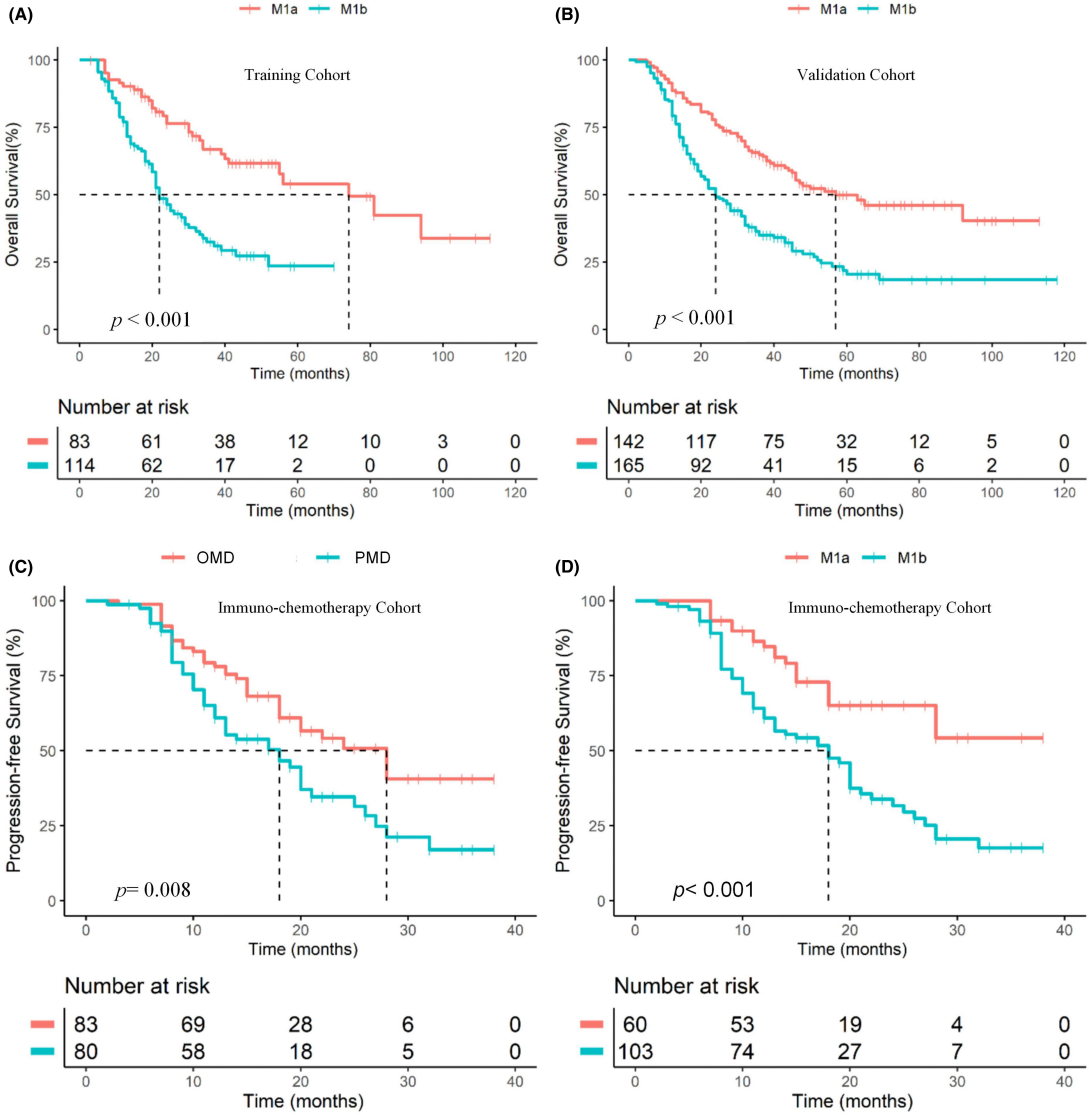
OS of M1a versus M1b subcategories in the training (A) and validation (B) cohorts, and PFS Stratified by OMD versus PMD (C) and by M1a versus M1b (D) in the immuno‐chemotherapy group. OS, overall survival; PFS, progression‐free survival; OMD, oligometastatic disease; PMD, polymetastatic disease.

Considering that the B and C groups had similar 3‐year OS and both did not benefit from LR‐RT, we combined them and revised the M1 classification proposal as M1a (OMD without liver metastases) and M1b (liver metastases or PMD). The 3‐year OS of M1a was better than that of M1b in both the training and validation cohorts (66.5% vs. 31.7%, p < 0.001; 64.9% vs. 35.0%, *p* < 0.001; Figure 3A,B). The C‐index of our refining M1 subcategory model for prognostic evaluation in the training and validation cohorts was 0.6258 and 0.6011, respectively, and time‐dependent receiver operating characteristic (ROC) curve analysis showed that the area under the curve (AUC) of the 3‐ and 5‐year OS in the training and validation cohorts were 0.691 and 0.659, and 0.766 and 0.644, respectively (Figure S5A,B). In the M1a group, patients who received LR‐RT had a higher 3‐year OS than those who did not (71.4% vs. 50.5%, *p* = 0.001), whereas those in the M1b group who received LR‐RT and did not receive LR‐RT showed no significant difference in OS (37.4% vs. 30.8%, *p* = 0.053, Figure S6).

### 3.6 Validation the usefulness of M1 subdivision in a separate immuno‐chemotherapy cohort

In order to verify the prognostic performance of the refining OMD model and M1 subcategories in era of immunotherapy, 163 patients with M1‐NPC who received chemotherapy plus anti‐PD‐1 in four centers were included. The median follow‐up time was 22 (range: 2–38) months (Table S2). Kaplan–Meier survival curves showed that patients with OMD had a superior median PFS compared to those with PMD (24 months vs. 17 months, *p* = 0.008, Figure 3C). In addition, patients with M1a had a significantly better median PFS than those with M1b (not reach vs. 17 months, *p* < 0.001, Figure 3D).

## 4 DISCUSSION

Current TNM‐8 has uniformly grouped M1‐NPC as one category, which lacks the ability to differentiate the prognosis of patients with M1‐NPC. Further subdivision of M1‐NPC has clinical value to better depict prognosis following contemporary treatment and may potentially guide treatment. Our study identified anatomic prognostic factors and proposed a clinical useful M1 subdivision in M1‐NPC. We found that metastasis involved ≤2 organs with ≤5 total metastatic lesions had the best distinction of OMD and PMD for both training and validation cohorts. Patients with liver metastasis also carried the worst outcomes versus other organ involvement. Based on OMD and liver metastases status, we proposed to subdivide M1 into M1a (OMD without liver metastases) and M1b (liver metastases or PMD). Our results also showed that patients in M1a could benefit from LR‐RT besides systemic therapy, whereas those in M1b could not. In addition, the subdivision of M1 into M1a and M1b could stratify the prognosis well in a separate cohort of M1‐NPC who received immuno‐chemotherapy.

Although the theory of OMD was refining in 1995,^24^ there remains no consensus definition in NPC. Although studies have found that the number of metastatic organs and lesions in NPC are important prognostic factors for M1‐NPC, few have systematically analyzed the prognostic value of the number of metastatic organs and lesions.^25^ The ESTRO‐ASTRO consensus also defines OMD as having 1–5 metastatic lesions but does not provide a specific definition for metastatic organs.^14^ By comparing the MVA model performance, we found that metastasis involved ≤2 metastatic organs with ≤5 metastatic lesions in total had the best ability to differentiate OMD versus PMD in our study. Our findings are consistent with the definition of OMD in metastatic colorectal cancer according to the ESMO consensus guidelines,^26^ although it is slightly different form a single‐center study from Singapore which showed that the OMD model with a single organ and ≤5 lesions had the best performance in OS prediction.^14^ However, their study also found that the OMD model with ≤2 organs and ≤5 metastatic lesions had similar performance to that of OMD with a single organ and ≤5 lesions (C‐index: 0.6152 and 0.6225). Recently, two retrospective studies from our center and other center found that the OMD model with ≤2 organs and ≤5 metastatic lesions could not only effectively evaluate the prognosis of patients with M1‐NPC, but could also help to screen populations benefit from LR‐RT.^4^, ^13^ Therefore, we believe that OMD (≤2 metastatic organs and ≤5 lesions) may be an appropriate definition in M1‐NPC.

Our results also confirmed that liver metastasis is an independent prognostic factor for M1‐NPC, which is consistent with previously published reports.^20^, ^27^ The C‐index of refining M1 subcategories model was higher than that of the OMD model in our study, suggesting the liver metastasis should also be considered in risk stratification of M1‐NPC. Importantly, the refining subcategories can also effectively distinguish beneficiaries from LR‐RT treatment. M1a patients can benefit from LR‐RT, whereas M1b patients do not. Although prospective and multiple retrospective studies have directly demonstrated that LR‐RT can provide survival benefits to patients with M1‐NPC,^18^, ^19^, ^28^, ^29^, ^30^ recent retrospective studies, including our previous study, have found that patients with OMD were the optimal candidates for LR‐RT.^4^, ^13^, ^20^, ^27^ However, current study confirmed that neither PMD patients nor OMD patients with liver metastases could benefit from LR‐RT. Local consolidative therapy of metastatic lesions has achieved a good therapeutic effect in many kinds of cancers,^9^, ^31^, ^32^ and our MVA results showed that local treatment of metastatic lesions did not improve the OS of NPC patients. This is probably because our local treatment were not consolidative treatments for all metastatic lesions. Currently, two ongoing phase III trials are investigating the role of consolidative by radiotherapy directed to all metastatic lesions in metastatic NPC (NCT05128201 and NCT04421469), and we are eagerly awaiting these results.

With the approval of anti‐PD‐1 mAbs for RM‐NPC in the first‐line setting, the era of immunotherapy for NPC is coming. It is important to determine whether refining OMD definition and M1 subcategory are still effective for M1‐NPC patients received immuno‐chemotherapy. As shown in our study, the refining OMD and M1 subcategories effectively depicted the prognosis of immune‐chemotherapy cohorts, indicating clinical relevance of the current definition of the OMD and M1 subcategories.

Several limitations of this study warrant discussion. First, the data were derived from multiple academic cancer centers in an endemic jurisdiction and whether the findings can be generalized to other patient populations remains to be determined. Second, despite our study showed that patients with liver metastasis or PMD status did not benefit from LR‐RT, the treatment is not randomly assigned and the findings should be validated. In addition, although the refined OMD definition and M1 subcategories in our study can effectively assess the PFS of patients treated with immuno‐chemotherapy, whether they can effectively assess OS remains to be determined. Owing to the short follow‐up time of the immunotherapy cohort, OS cannot be accurately assessed at present, and subsequent long‐term follow‐up verification remains necessary. Finally, we did not include EBV DNA in the M1 subdivision since this is an anatomic grouping since this is an anatomic grouping. It is conceivable that EBV DNA has a potential to be included for prognostic grouping to enhance outcome prediction after consistency in EBV DNA testing is demonstrated.^27^, ^28^

## 5 CONCLUSION

The definition of ≤2 metastatic organs and ≤5 metastatic lesions is the most appropriate separation of OMD versus PMD for M1‐NPC in our study. Liver metastasis is also a strong adverse prognostic factor. Patients with OMD without liver metastasis have better OS and can be considered as M1a while the remaining as M1b. The M1a (OMD without liver metastasis) and M1b (liver metastasis or PMD) subdivision provides better prognostic evaluation for patients with M1‐NPC receiving immuno‐chemotherapy, and have the potential to screen the population regarding who will gain the greatest benefit from LR‐RT. Further external cohorts, especially multicenter studies, are warranted to validate the prognostic value of the refining M1 subcategories.

## AUTHOR CONTRIBUTIONS

**Tian‐Zhu Lu:** Conceptualization (equal); formal analysis (equal); funding acquisition (equal); writing – original draft (equal); writing – review and editing (equal). **Fu‐juan Zeng:** Conceptualization (equal); data curation (equal); formal analysis (equal); writing – original draft (equal); writing – review and editing (equal). **Yu‐Jun Hu:** Conceptualization (equal); data curation (equal); formal analysis (equal); writing – original draft (equal); writing – review and editing (equal). **Min Fang:** Data curation (equal); investigation (equal); writing – review and editing (equal). **Fang‐yan Zhong:** Data curation (equal); formal analysis (equal); methodology (equal); writing – review and editing (equal). **Bi‐juan Chen:** Investigation (equal); resources (equal). **Hao Zhang:** Investigation (equal); resources (equal). **Qiao‐juan Guo:** Data curation (supporting); investigation (supporting); writing – review and editing (supporting). **Jian‐ji Pan:** Project administration (equal); supervision (equal); writing – review and editing (equal). **Xiao‐chang Gong:** Data curation (equal); formal analysis (equal); resources (equal); supervision (equal). **Shao Hui Huang:** Supervision (equal); writing – review and editing (equal). **Zhao‐hui Liao:** Resources (equal); supervision (equal). **Yunfei Xia:** Conceptualization (equal); project administration (equal); writing – review and editing (lead). **Jin‐gao Li:** Conceptualization (equal); data curation (equal); funding acquisition (equal); project administration (equal); supervision (equal); writing – original draft (lead); writing – review and editing (lead).

## FUNDING INFORMATION

This work was supported by the National Natural Science Foundation of China (grant 82103478, 82160710), the Open Fund for Scientific Research of Jiangxi Cancer Hospital (grant 2021 J13, 2021K01), Non‐profit Central Research Institute Fund of Chinese Academy of Medical Sciences (grant 2020‐PT320‐004), and the Jiangxi Province Key R&D Program (Key Program) (grant 20232BBG70025).

## CONFLICT OF INTEREST STATEMENT

All authors have no conflicts of interest to declare.

## ETHICS APPROVAL STATEMENT

The study was approved institution's ethics review board of Jiangxi Cancer Hospital (No. 2021ky164).

## Supporting information


Figure S1.

## Data Availability

The data that support the findings of this study are available on request from the corresponding author.
