# Supplementary material for: Anatomic prognostic factors and their potential roles in refining M1 classification for de novo metastatic nasopharyngeal carcinoma
Source: Cancer Med. 2023 Dec 11;12(24):22091–102. doi: 10.1002/cam4.6816 (PMC10757129; doi:10.1002/cam4.6816)
Supplement: Supplementary file 1 — Figure S1. [file CAM4-12-22091-s001.docx]

**Supplementary Material**

**1. Supplementary Figures**

**Supplementary Fig. 1.** Flow chart of patients included in analyses.

**Supplementary Fig. 2**. The Overall survival for different OMD models in the training cohort. Model 1, one metastatic lesion (A); model 2, one metastatic organ and ≤3 metastatic lesions (B); model 3, one metastatic organ and ≤5 metastatic lesions (C); model 4, ≤2 metastatic organs and ≤3 metastatic lesions (D); model 6, (≤3 metastatic organs and ≤5 metastatic lesions (E).

Abbreviations: OS, Overall survival; OMD, oligometastatic disease; PMD, polymetastatic disease.

**Supplementary Fig. 3**. The Overall survival for different OMD models in the validation cohort. Model 1, one metastatic lesion (A); model 2, one metastatic organ and ≤ 3 metastatic lesions (B); model 3, one metastatic organ and ≤ 5 metastatic lesions (C); model 4, ≤ 2 metastatic organs and ≤ 3 metastatic lesions (D); model 6, (≤ 3 metastatic organs and ≤5 metastatic lesions (E).

Abbreviations: OS, Overall survival; OMD, oligometastatic disease; PMD, polymetastatic disease.

**Supplementary Fig. 4**. The Overall survival for liver metastases in the training (A) and validation cohorts (B).

Abbreviations: OS, Overall survival.

**Supplementary Fig 5.** The time-dependent receiver operating characteristic curves of OS for proposed M1b.

Abbreviations: OS, Overall survival.

**Supplementary Fig. 6.** The Overall survival for patients received LR-RT or not in OMD without liver metastases

Abbreviations: OS, Overall survival; OMD, oligometastatic disease; LRRT: locoregional radiotherapy.

**2. Supplementary Tables**

2.1 Supplementary Table 1. Univariable analysis of OS in dmNPC patients of training (n=197) and validation cohorts (n=307).

2.2 Supplementary Table 2. Patient characteristics in immune checkpoint inhibitors cohort (n=163).

1. **Supplementary Method**

***Treatment***

Patients were treated with IMRT at a cumulative dose of 66-70 Gy/30-35 fractions to the planning target volume (PTV) of the primary gross tumor volume (GTVnx) and 64-70 Gy/30-35 fractions to the PTV of the involved lymph nodes (GTVnd). The prescribed doses were 60-66 Gy/30-35 fractions to the PTV of the high-risk clinical target volume (CTV1) and 54-56 Gy/30-35 fractions to the PTV of the low-risk clinical target volume (CTV2). All patients were treated with one fraction daily, 5 days per week. The chemotherapy regimens comprised TP, PF, TPF, and GP. The TP regimen consisted of docetaxel 75 mg/m2/paclitaxel 135-175 mg/m2 on day 1, and cisplatin/nedaplatin 80-100mg/m2 on day 1. The PF regimen comprised cisplatin/nedaplatin 80-100 mg/m2 on day 1, and 5-Fu 800-1000 mg/m2/d civ 120h. The TPF regimen consisted of docetaxel 60-75 mg/m2/paclitaxel 135-175 mg/m2 on day 1, cisplatin/nedaplatin 60-75 mg/m2 on day 1, and 5-Fu 500-750 mg/m2/d civ 120h. The GP regimen comprised cisplatin/nedaplatin 80-100 mg/m2 on day 1 and gemcitabine 1000 mg/m2 on day 1 and day 8. In some cases, cisplatin/nedaplatin was replaced with oxaliplatin (85-130 mg/m2), lobaplatin (30 mg/m2) or carboplatin (AUC = 5).

**Management of metastatic lesions**

For metastases suitable for local treatment, the attending physician and patient jointly formulated a treatment strategy, e.g. radiotherapy and radiofrequency ablation. The radiotherapy dose was determined according to the target size and location of the lesion by IMRT or conventional radiotherapy, but generally ranged from 30 to 54 Gy, and the commonly used radiation doses are 2 Gy/F×20F, 2 Gy/F×25F, 3 Gy/F×10F, etc. In the event of spinal cord compression or pain, symptom reduction therapy is also performed for the metastases, and the specific dose is determined by the clinician's assessment.

**Supplement Figures**

**Supplementary Fig. 1**. Flow chart of patients included in analyses.


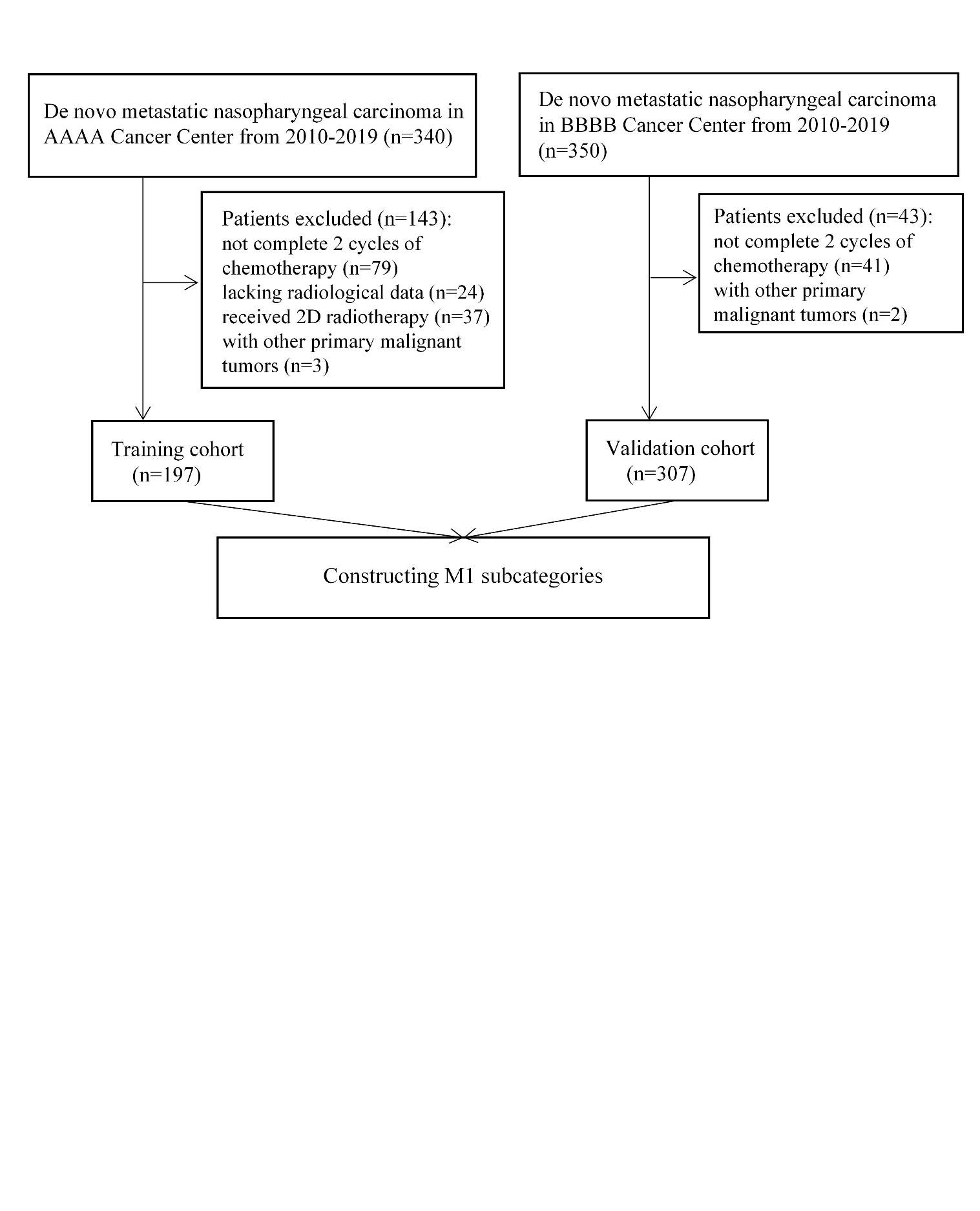


**Supplementary Fig. 2.** The Overall survival for different OMD models in the training cohort.. Model 1, one metastatic lesion (A); model 2, one metastatic organ and ≤3 metastatic lesions (B); model 3, one metastatic organ and ≤5 metastatic lesions (C); model 4,≤2 metastatic organs and ≤3 metastatic lesions (D); model 6, (≤3 metastatic organs and ≤5 metastatic lesions (E).


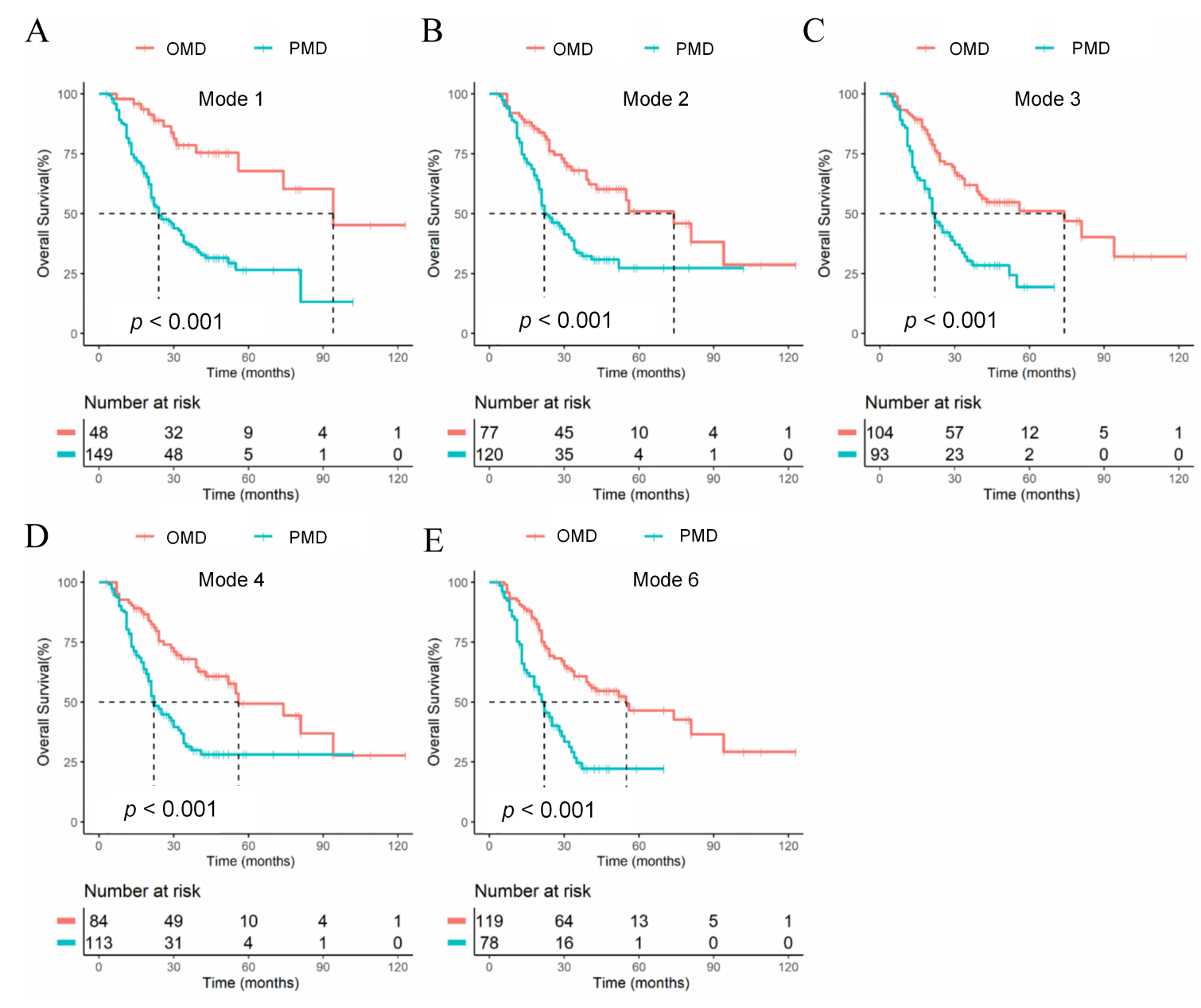


Abbreviations: OS, Overall survival; OMD, oligometastatic disease; PMD, polymetastatic disease.

**Supplementary Fig. 3**. The Overall survival for different OMD models in the validation cohort. Model 1, one metastatic lesion. Model 1, one metastatic lesion (A); model 2, one metastatic organ and ≤ 3 metastatic lesions (B); model 3, one metastatic organ and ≤ 5 metastatic lesions (C); model 4,≤ 2 metastatic organs and ≤ 3 metastatic lesions (D); model 6, (≤ 3 metastatic organs and ≤5 metastatic lesions (E).


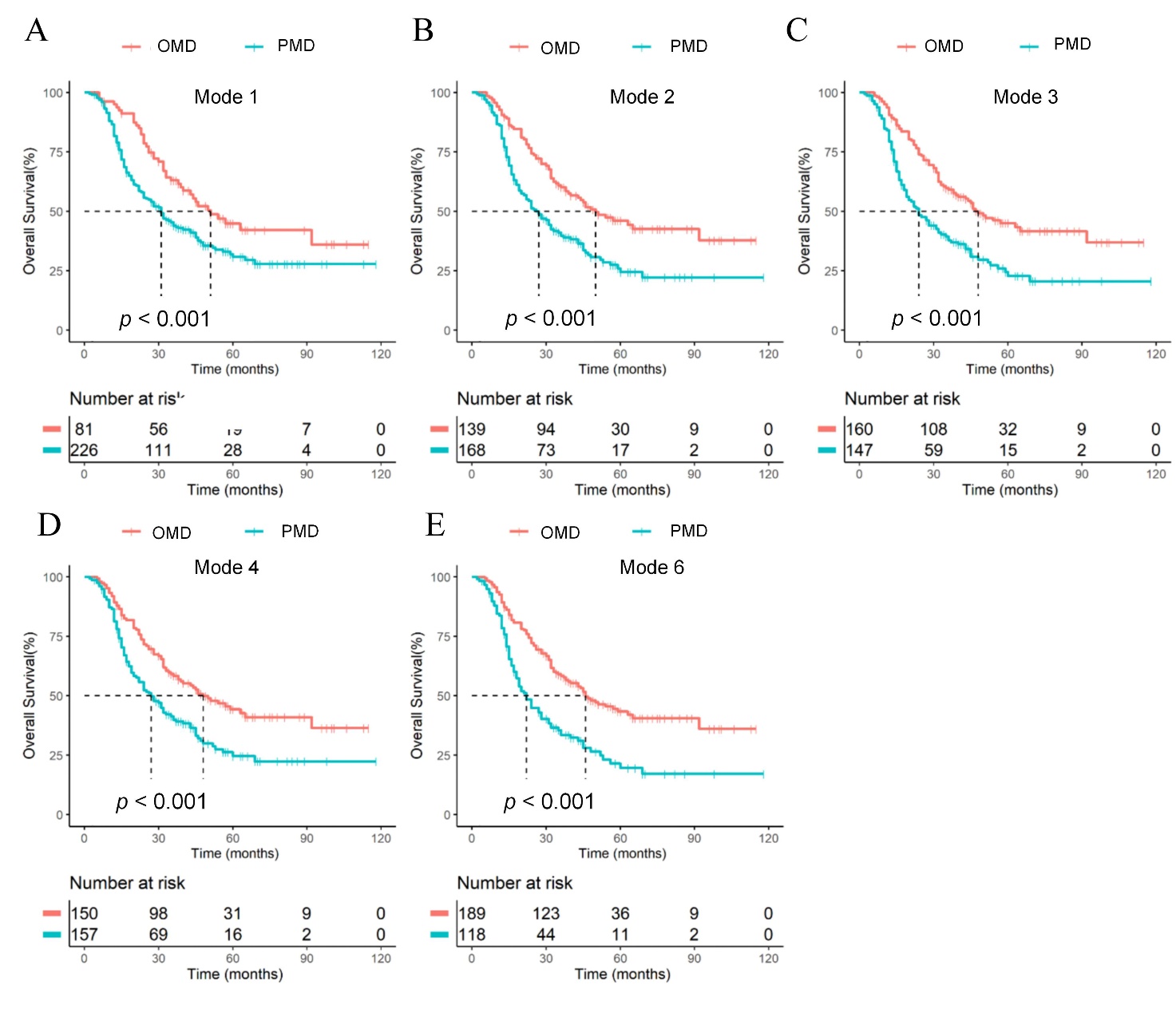


Abbreviations: OS, Overall survival; OMD, oligometastatic disease; PMD, polymetastatic disease.

**Supplementary Fig. 4**. The Overall survival for liver metastases in the training (A) and validation cohorts (B)
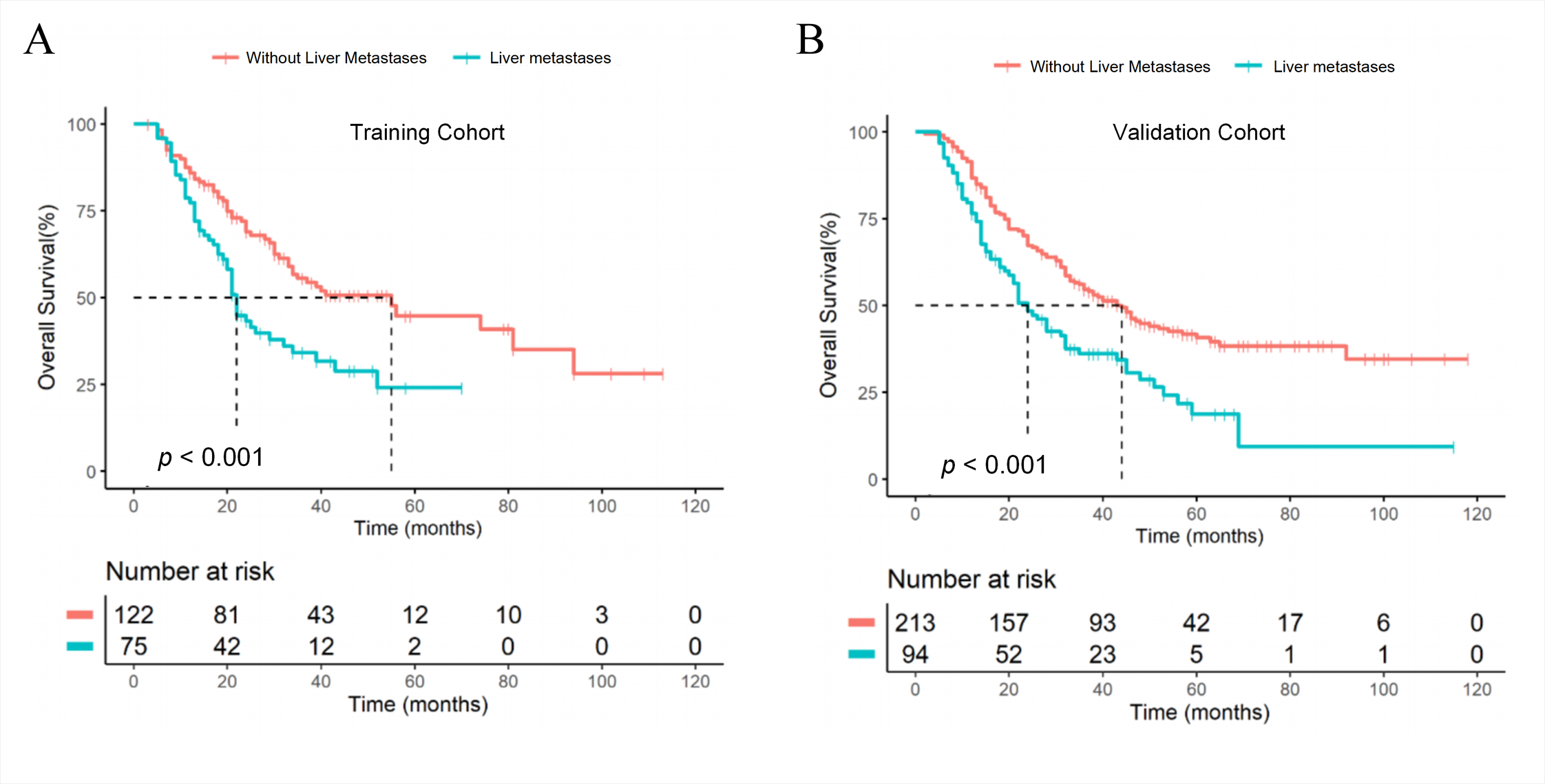


Abbreviations: OS, Overall survival.

**Supplementary Fig. 5.** The time-dependent receiver operating characteristic curves of OS for proposed M1b.


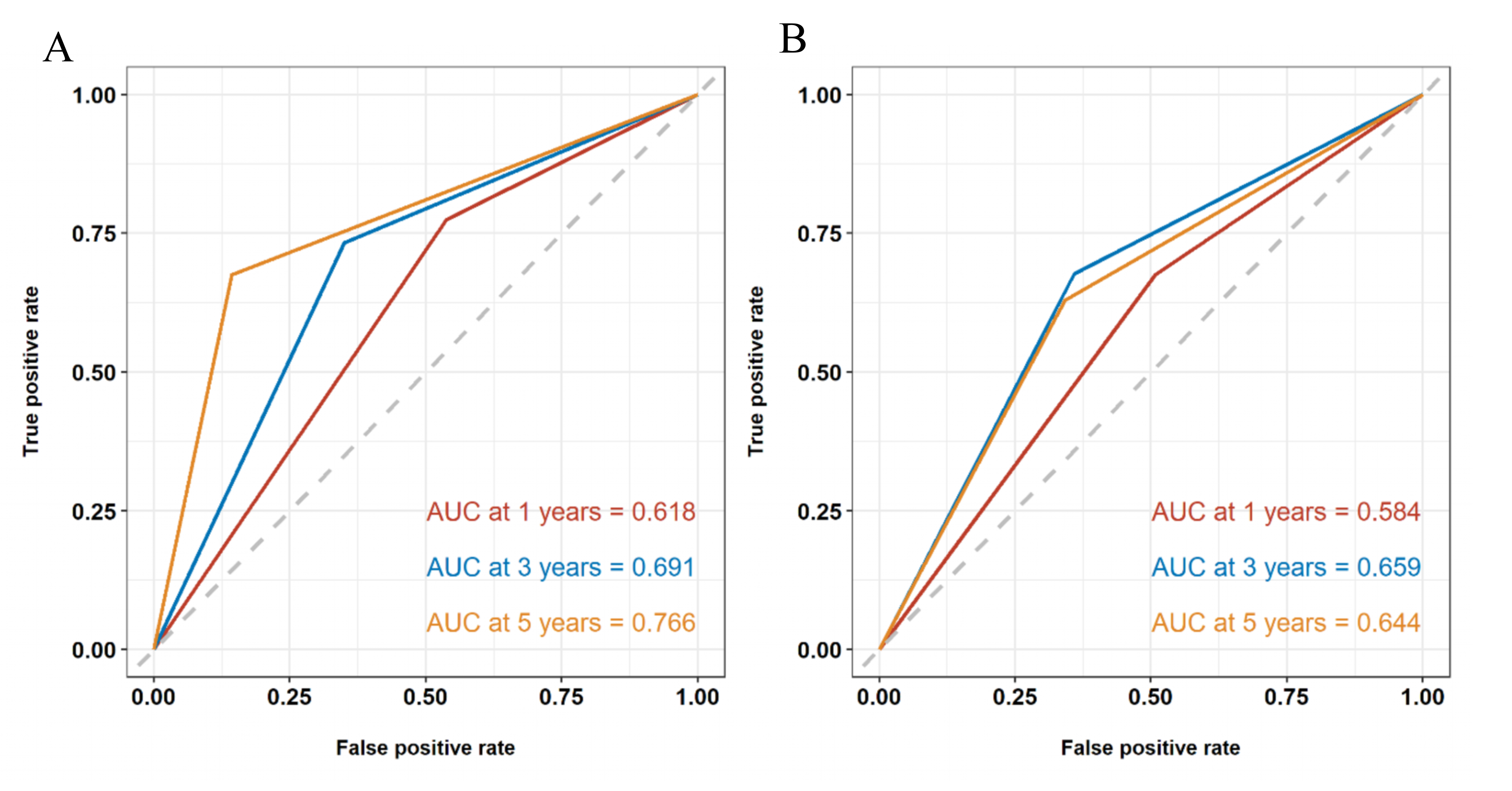


Abbreviations: OS, Overall survival.

**Supplementary Fig. 6.** The Kaplan-Meier curves of OS for patients who received LRRT or not in OMD without liver metastases.


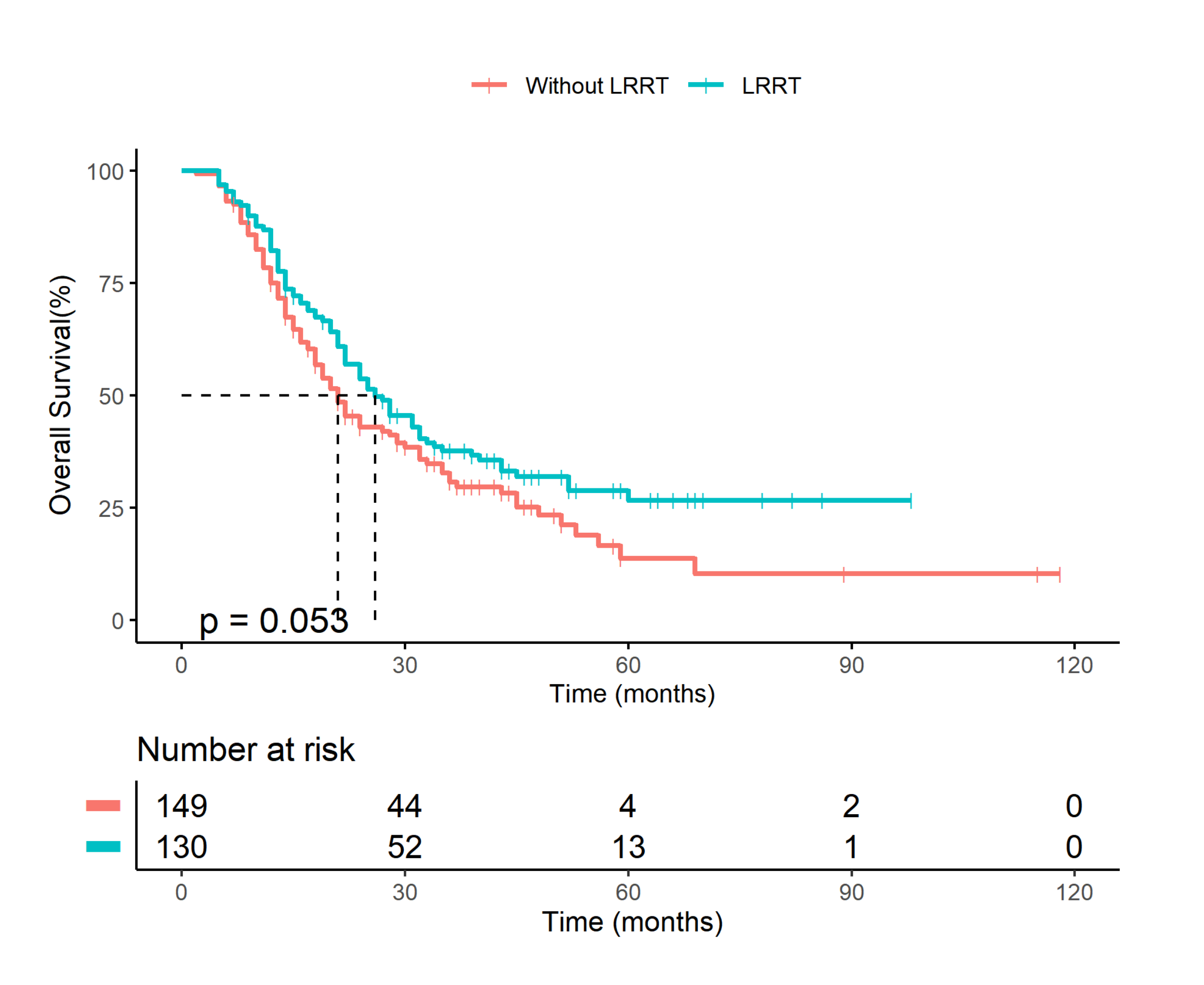


Abbreviations: OS, Overall survival; OMD, oligometastatic disease; LRRT: locoregional radiotherapy.

**Supplementary Tables**

**Supplementary Table 1**. Univariable analysis of OS in dmNPC patients of training (n=197) and validation cohorts (n=307).

| Variables | Training Cohort | |  | Validation Cohort | |
| --- | --- | --- | --- | --- | --- |
|  | HR (95%CI) | p |  | HR (95%CI) | p |
| Gender (male vs. Female) | 0.76 (0.44-1.31) | 0.316 |  | 0.73 (0.49-1.11) | 0.144 |
| Age (≤median vs. ＞median) | 0.99 (0.67-1.45) | 0.955 |  | 1.14 (0.85-1.52) | 0.379 |
| ECOG (0-1 vs. 2) | 1.71 (0.69-4.23) | 0.245 |  | 1.058 (0.47-2.39) | 0.891 |
| T categories (T1-2 vs. T3-4) | 1.15 (0.77-1.70) | 0.495 |  | 0.84 (0.62-1.14) | 0.252 |
| N categories (N0-1 vs. N2-3) | 1.15 (0.68-1.94) | 0.596 |  | 1.40 (0.97-2.02) | 0.069 |
| Bone metastases (No vs. Yes) | 1.45 (0.95-2.19) | 0.084 |  | 0.92 (0.67-1.26) | 0.589 |
| Liver metastases (No vs. Yes) | 1.83 (1.24-2.72) | 0.003 |  | 1.75 (1.29-2.36) | <0.001 |
| Lung metastases (No vs. Yes) | 0.79 (0.49-1.27) | 0.327 |  | 0.89 (0.63-1.26) | 0.504 |
| Other metastases (No vs. Yes) | 1.31 (0.77-2.23) | 0.327 |  | 1.33 (0.95-1.87) | 0.098 |
| LR-RT (No vs. Yes) | 0.44 (0.30-0.66) | <0.001 |  | 0.54 (0.35-0.83) | <0.001 |
| Local treatment for metastatic lesions (No vs. Yes) | 0.73 (0.44-1.14) | 0.217 |  | 0.54 (0.35-0.83) | 0.005 |
| Chemotherapy (＜6 cycles vs. ≥6 cycles) | 0.95 (0.57-1.56) | 0.827 |  | 0.87 (0.64-1.17) | 0.357 |
| EBV DNA (Low vs. High) | 1.76 (1.07-2.88) | 0.025 |  | 1.70 (1.21-2.36) | 0.002 |
| LDH (Normal vs. Abnormal*) | 2.11 (1.41-3.14) | <0.001 |  | 1.39 (1.03-1.87) | 0.029 |
| No. of the metastatic organs (1-2 vs. ≥3) | 1.97 (1.31-2.95) | 0.001 |  | 1.53 (1.13-2.06) | 0.006 |
| No. of the metastatic lesion (≤5 vs. ＞5) | 2.56 (1.72-3.81) | <0.001 |  | 2.02 (1.51-2.71) | <0.001 |

Abbreviations: dmNPC, *de novo* metastatic nasopharyngeal carcinoma; LR-RT, locoregional radiotherapy; LDH, Lactate dehydrogenase; EBV DNA, Epstein-Barr virus deoxyribonucleic acid; *Abnormal, >250U/L.

**Supplementary Table 2**. Patient characteristics in immune checkpoint inhibitors cohort (n=163).

| Variables | N (%) |
| --- | --- |
| Age, median (IQR) (years) | 47 (38−56) |
| Sex |  |
| Male | 124 (76.1) |
| Female | 39 (23.9) |
| Histology |  |
| Keratinizing | 1 (0.6) |
| Nonkeratinizing | 162 (99.4) |
| Tumor category |  |
| T1 | 9 (5.5) |
| T2 | 13 (8.0) |
| T3 | 82 (50.3) |
| T4 | 59(36.2) |
| Node category |  |
| N1 | 24 (14.7) |
| N2 | 51 (31.3) |
| N3 | 88 (54.0) |
| Liver metastases |  |
| No | 130 (79.7) |
| Yes | 63 (39.3) |
| Bone metastases |  |
| No | 59 (36.2) |
| Yes | 104 (63.8) |
| Lung metastases |  |
| No | 117 (71.8) |
| Yes | 46 (28.2) |
| Other site metastases |  |
| No | 122 (74.8) |
| Yes | 41 (25.2) |
| No. of metastatic sites |  |
| ≤ 2 | 146 (89.6) |
| > 2 | 17 (10.4) |
| No. of metastatic lesions |  |
| ≤ 5 | 84 (51.5) |
| > 5 | 79 (48.5) |
| LDH (U/L)* |  |
| Normal | 114 (70.0) |
| Abnormal | 49 (30.0) |
| EBV DNA** |  |
| Undetectable | 13 (8.0) |
| Detectable | 145 (89.0) |
| NA | 5 (3.0) |
| Cycles of first-line chemotherapy |  |
| < 6 | 48 (29.4) |
| ≥ 6 | 115 (70.6) |

Abbreviations: OMD, oligometastatic disease; LRRT, locoregional radiotherapy; EBV DNA, Epstein-Barr virus deoxyribonucleic acid; *Abnormal threshold, >250U/L; **: Detectable thresholds: 0 copy/mL.
